# Towards universal comparability of pericoronary adipose tissue attenuation: a coronary computed tomography angiography phantom study

**DOI:** 10.1007/s00330-022-09274-5

**Published:** 2022-12-06

**Authors:** Dominik Etter, Geoff Warnock, Frederic Koszarski, Tilo Niemann, Nidaa Mikail, Susan Bengs, Ronny R. Buechel, Philipp Kaufmann, Cathérine Gebhard, Alexia Rossi

**Affiliations:** 1 Department of Nuclear Medicine, University Hospital Zurich, Raemistrasse 100, 8091 Zurich, Switzerland; 2 Center for Molecular Cardiology, University of Zurich, 8952 Schlieren, Switzerland; 3 Department of Radiology, Kantonsspital Baden, 5400 Baden, Switzerland

**Keywords:** Adipose tissue, Computed tomography angiography, Coronary arteries, Image reconstruction

## Abstract

**Objectives:**

Different computed tomography (CT) scanners, variations in acquisition protocols, and technical parameters employed for image reconstruction may introduce bias in the analysis of pericoronary adipose tissue (PCAT) attenuation derived from coronary computed tomography angiography (CCTA). Therefore, the aim of this study was to establish the effect of tube voltage, measured as kilovoltage peak (kVp), and iterative reconstruction on PCAT mean attenuation (PCAT_MA_).

**Methods:**

Twelve healthy ex vivo porcine hearts were injected with iodine-enriched agar-agar to allow for ex vivo CCTA imaging on a 256-slice CT and a dual-source CT system. Images were acquired at tube voltages of 80, 100, 120, and 140 kVp and reconstructed by using both filtered back projection and iterative reconstruction algorithms. PCAT_MA_ was measured semi-automatically on CCTA images in the proximal segment of coronary arteries.

**Results:**

The tube voltage showed a significant effect on PCAT_MA_ measurements on both the 256-slice CT scanner (*p* < 0.001) and the dual-source CT system (*p* = 0.013), resulting in higher attenuation values with increasing tube voltage. Similarly, the use of iterative reconstructions was associated with a significant increase of PCAT_MA_ (256-slice CT: *p* < 0.001 and dual-source CT: *p* = 0.014). Averaged conversion factors to correct PCAT_MA_ measurements for tube voltage other than 120 kVp were 1.267, 1.080 and 0.947 for 80, 100, and 140 kVp, respectively.

**Conclusion:**

PCAT_MA_ values are significantly affected by acquisition and reconstruction parameters. The same tube voltage and reconstruction type are recommended when PCAT attenuation is used in multicenter and longitudinal studies.

**Key Points:**

*• The tube voltage used for CCTA acquisition affects pericoronary adipose tissue attenuation, resulting in higher attenuation values of fat with increasing tube voltage.*

*• Conversion factors for pericoronary adipose tissue attenuation values could be used to adjust for differences in attenuation between scans performed at different tube voltages.*

*• In longitudinal CCTA studies employing pericoronary adipose tissue attenuation as imaging endpoint, it is recommended to maintain tube voltage and image reconstruction type constant across serial scans.*

**Supplementary Information:**

The online version contains supplementary material available at 10.1007/s00330-022-09274-5.

## Introduction

Vascular inflammation is a key factor in the progression of atherosclerosis, potentially resulting in plaque rupture and adverse cardiovascular events [1]. Therefore, its early detection may allow targeted therapies in patients with coronary artery disease, preventing future heart attacks [2]. A recent study reported that the presence of vascular inflammation might lead to the release of inflammatory mediators into the local pericoronary adipose tissue (PCAT), inducing local lipolysis and inhibiting adipogenesis [3]. This is associated with an increased water content of PCAT, which can be detected non-invasively by coronary computed tomography angiography (CCTA) as an increased attenuation (Hounsfield unit, HU) surrounding the affected vessel [3].

A growing body of evidence demonstrated that PCAT attenuation is a useful diagnostic and prognostic marker of coronary inflammation in patients with coronary artery disease [4–7]. Despite this, as with other novel quantitative imaging biomarkers, standardization in image acquisition, analysis, and interpretation, together with the identification of normal values, are required before PCAT may be implemented as a clinical tool. It is well known that computed tomography (CT) attenuation is sensitive to, among other factors, scanner type, tube voltage, type of image reconstruction, and filter kernel [8–10]. Nevertheless, to date, only limited data are available on the effect of these acquisition and reconstruction parameters on PCAT attenuation in healthy coronary arteries [11]. This is an important issue for longitudinal and multicenter studies where the proper design of scan and image reconstruction protocols is of paramount importance to ensure the collection of accurate and comparative data. Therefore, the aim of the current study was twofold: (1) to evaluate the influence of tube voltage, measured as kilovoltage peak (kVp), and image reconstruction algorithms on PCAT attenuation values, and (2) to evaluate the magnitude of the effect of tube voltage on PCAT attenuation by calculating specific conversion factors for PCAT measurements according to different kVp, commonly used in clinical routine, by using ex vivo porcine hearts.

## Materials and methods

### Porcine hearts

Fresh adult porcine hearts were ordered through the Veterinary Department of Zürich, Switzerland, and obtained directly from the slaughterhouse of Zürich (Schlachtbetrieb Zürich AG, Switzerland). As the pigs were destined to be slaughtered, no further ethical approval was necessary. A total of 12 healthy hearts were used for the purpose of the study.

The hearts were washed, the pericardium was removed, and the aorta was severed after the ramification of the left subclavian artery. The left ventricle (LV) was filled through the aorta with 1% agar-agar and 9 mg/mL iodine (Iodixanol, GE Healthcare) solution at 40 °C. After 5 min of curing time, the same agar solution was injected into the main coronary arteries, either directly via their origin or from their origin and distal end simultaneously, until the coronary artery was visually filled.

**Fig. 1:**
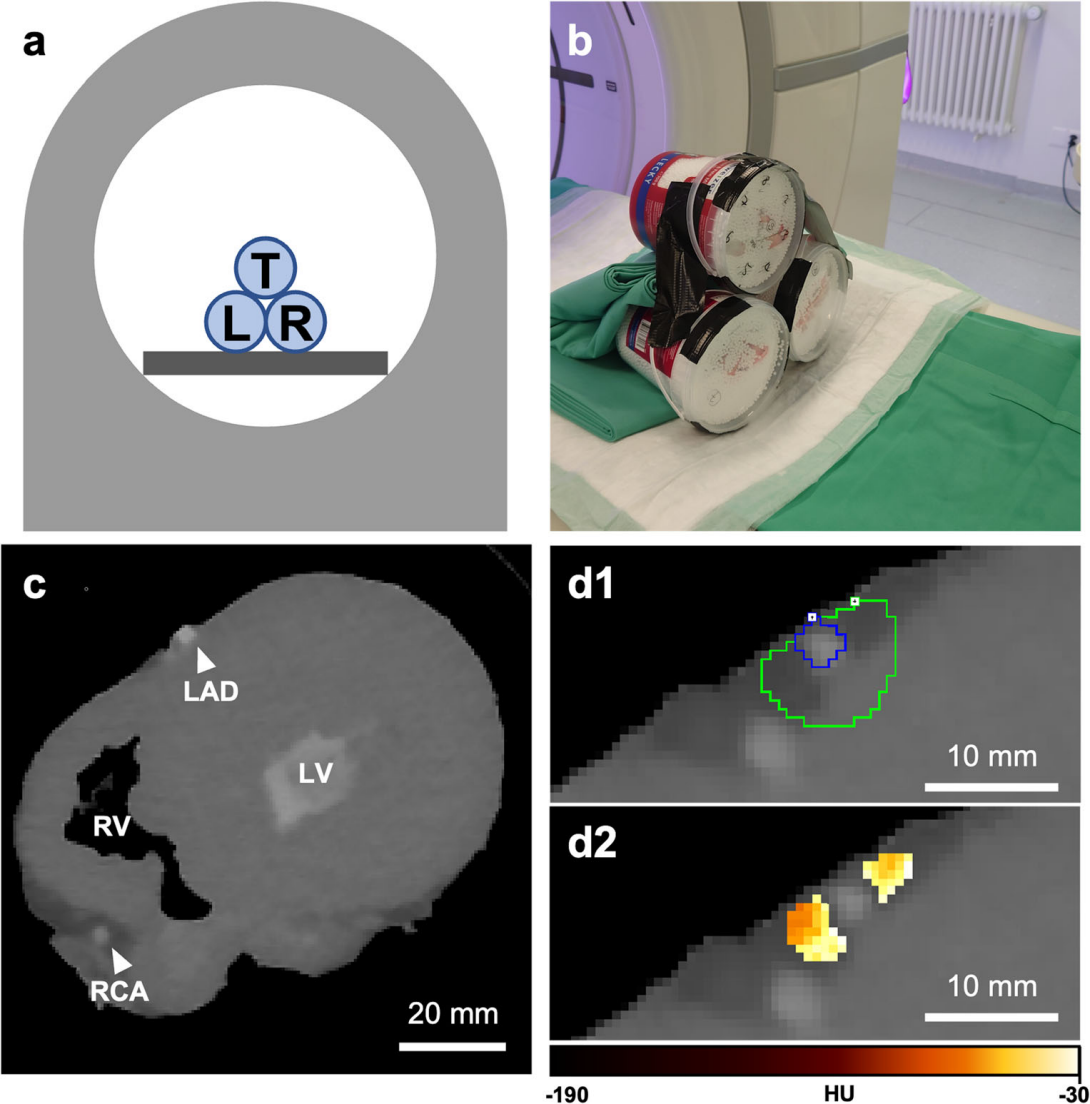
Experimental set-up and image analysis. **a** Schematic representation of the experimental set-up showing the hearts positioned to resemble a pyramidal stack: top row (centered position, T), left bottom row (L), and right bottom row (R). **b** Arrangement of three porcine hearts in containers used for imaging. **c** CT short-axis view of the porcine heart. The LV and the coronary arteries are filled with 1% agar-agar and 9 g/mL iodine solution. **d1** Volume of interest in a transaxial view of the LAD. The blue outline indicates the vessel lumen while the green one marks the surrounding volume evaluated for PCAT attenuation. **d2** PCAT phenotyping in transaxial view. The heatmap corresponds to the established HU range of adipose tissue (**d2**). *Abbreviations: CT, computed tomography; HU, Hounsfield unit; L, left; LAD, left anterior descending artery; LV, left ventricle; PCAT, pericoronary adipose tissue; R, right; RCA, right coronary artery; RV, right ventricle: T, top*

After curing, the hearts were placed inside cylindrical radiolucent plastic containers and expanded polystyrene beads were used to fill the surrounding volume as shown in Fig. 1a, b. The preparation was reproducible and constituted a clean and easy-to-handle ex vivo heart phantom with contrast-enriched coronaries as demonstrated in Fig. 1c.

### CCTA data acquisition

CCTA scans were performed within 4 h from the phantom’s preparation to avoid loss of contrast. Nine hearts were scanned on a 256-slice CT scanner (Revolution CT, GE Healthcare) using prospectively electrocardiogram (ECG)–triggered axial mode whereas the remaining three hearts were scanned on a dual-source CT (Siemens SOMATOM Drive, Siemens Healthineers) by retrospectively ECG-gated acquisition. On both systems, scans were performed at 80, 100, 120, and 140 kVp with a total exposure of 56 milliampere-seconds (mAs) and 50 mAs for the 256-slice CT and dual-source CT, respectively. A simulated ECG signal triggering was used for image acquisition on both scanners.

All scans were reconstructed using pure filtered back projection (FBP) and iterative reconstruction (IR) algorithms with three iterations [adaptive statistical iterative reconstruction (ASIR-V) for 256-slice CT and advanced modeled iterative reconstruction (ADMIRE) for dual-source CT]. For the 256-slice CT scanner, *standard* kernel was used for FBP images and *standard2* kernel for IR images whereas for the dual-source CT system, all datasets were reconstructed by using the Bv38 kernel. Images were reconstructed either in a 512 × 512 × 256 voxel matrix at voxel sizes of 0.5859 × 0.5859 × 0.6 mm^3^ (256-slice CT) or 512 × 512 × 261 voxel matrix at voxel sizes of 0.5352 × 0.5352 × 0.625 mm^3^ (dual-source CT).

### Image post-processing and evaluation

PCAT attenuation measurements were performed using a semi-automated workflow in PMOD 4.203 (PMOD Technologies LLC). All visible coronary arteries were manually traced starting at their origin for up to 40 mm or for the total length available by using the images acquired at 120 kVp and reconstructed with IR. The tissue within a radial distance from the artery equal to the vessel diameter was considered perivascular adipose tissue returning a tube-like 3-dimensional (3D) volume of interest (VOI) along each vessel, as previously reported [3, 7]. Within this 3D VOI, the adipose tissue was defined as all voxels with an attenuation ranging between − 190 and − 30 HU, as demonstrated in Fig. 1d1, d2. The 3D VOI obtained from the 120-kVp images was then transferred to all remaining co-registered datasets acquired at 80, 100, and 140 kVp, and the PCAT mean attenuation (PCAT_MA_) was calculated.

### Conversion factors

To correct for differences in attenuation between scans performed at tube voltages other than 120 kVp, conversion factors (*k*_*i*_) for PCAT_MA_ were calculated from IR datasets using the following formula:

1 $$ {k}_i=\frac{PCAT_{MA,i}}{PCAT_{MA,120}} $$

where *PCAT*_*MA*, *i*_ is the estimated PCAT_MA_ at tube voltage *i* ∈ [80, 100, 140]. The conversion of the attenuation values estimated at tube voltage *i* was then achieved by applying:

2 $$ {I}_{120}=\frac{I_i}{k_i} $$

where *I*_*i*_ is the attenuation at tube voltage *i*.

### Data processing and statistics

Data processing and statistical analyses were performed in Python 3.7.11 under Conda 4.10.3 [12, 13]. CT attenuation values are presented as mean ± standard error whereas conversion factors are shown as mean ± standard deviation. The standard deviation for each conversion factor was calculated according to the method reported in [14]. Repeated-measures ANOVA was performed to detect significant differences in PCAT_MA _between different tube voltages. Post hoc comparisons were performed using paired-sample *t* testing. All tests were two-sided and *α* was set at 0.05.

## Results

### Heart phantoms

Filling the LV and the coronary arteries with contrast-enriched agar provided sufficient visibility of the vessel lumen in the reconstructed images. All detectable coronary arteries were traced on the 120-kVp images since the methodology was verified originally at this tube voltage. In all 12 prepared hearts, tracing of at least one coronary artery was possible. PCAT_MA_ measurements were feasible in 21/36 (58.3 %) of all prepared vessels. Mean VOI size was 1406 ± 62.08 voxels. The main reason preventing the measurement of PCAT_MA_ was the loss of injected agar solution due to either leaks or para-injection of the agar solution during preparation. Both resulted in a lack of contrast between the vessel’s lumen and the surrounding tissue, hence preventing proper vessel tracing.

**Fig. 2:**
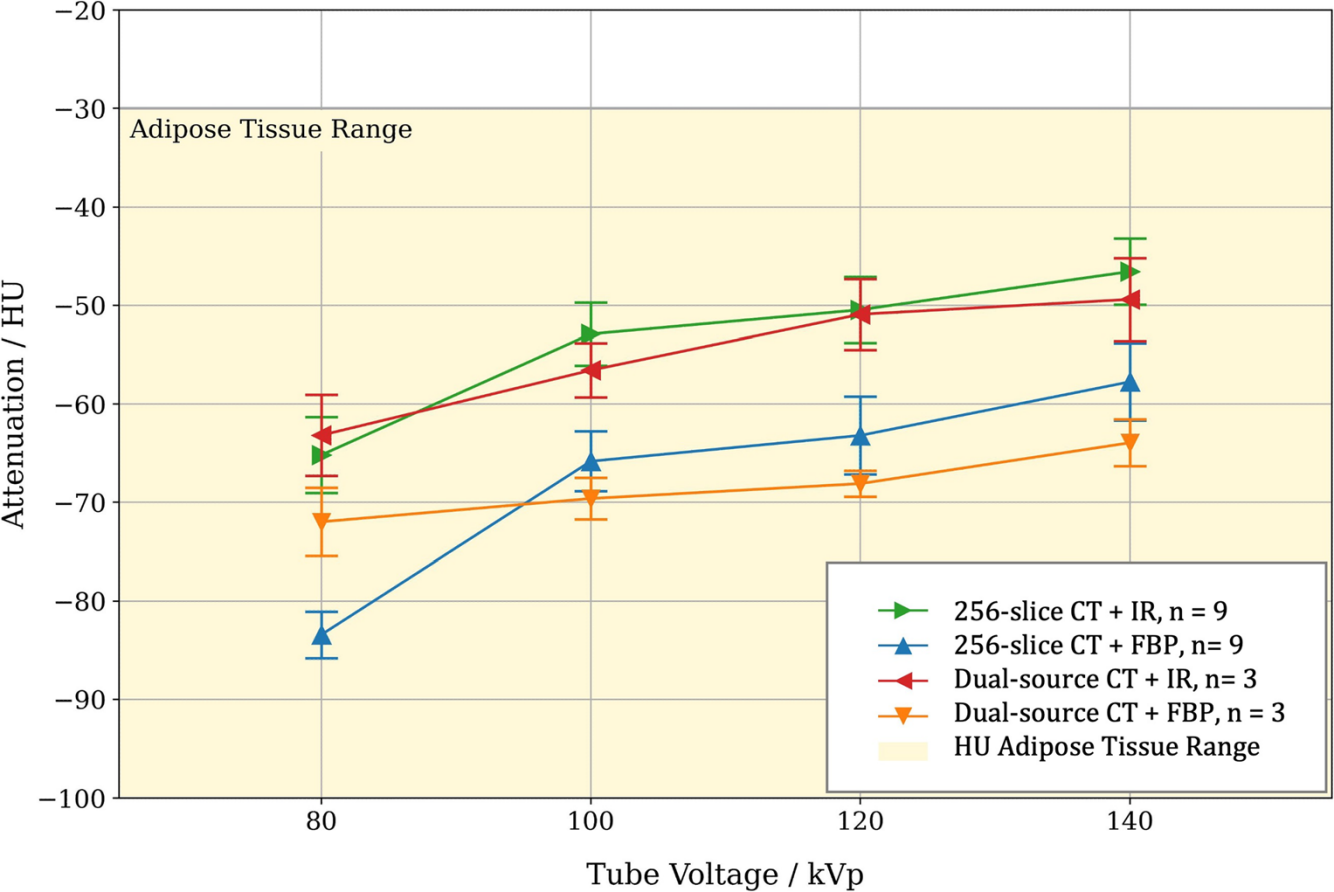
Effect of kVp and image reconstruction on PCAT_MA_ in porcine hearts. For both CT manufacturers, PCAT_MA_ increased with increasing values of tube voltages. The implementation of IR resulted in higher values of PCAT_MA_ as compared to FBP for each value of tube voltage. The yellow background indicates the HU range corresponding to adipose tissue. Means and standard errors are reported. *Abbreviations: CT, computed tomography; FBP, filtered back projection; HU, Hounsfield unit; IR, iterative reconstruction; kVp, kilovoltage peak; PCAT*_*MA*_*, pericoronary adipose tissue mean attenuation*

The measurements shown in Fig. 2 demonstrate the effect of increasing tube voltage on PCAT_MA_ values. For both 256-slice CT scanner and dual-source CT system, the applied tube voltage led to a statistically significant difference in PCAT_MA_ (tube voltage effect: *p* < 0.001 for 256-slice CT and *p* = 0.013 for dual-source CT). The highest PCAT_MA_ values were measured at 140 kVp and the lowest ones at 80 kVp. Additionally, a significant difference in PCAT_MA_ values was observed between IR and FBP reconstructed images for both 256-slice CT (group effect, *p* < 0.001) and dual-source CT (group effect, *p* = 0.014) scanners. In particular, for both manufacturers, the use of IR resulted in a higher PCAT_MA_ as compared to FBP. No significant difference was found between the two manufacturers for measurements based on the same type of reconstruction.

### Conversion factors

The conversion factors and their corresponding standard deviations for tube voltages of 80, 100, and 140 kVp derived by using 120 kVp as a reference are shown in Table 1. In our experimental setting, the different positions of the hearts inside the phantom resulted in slightly different conversion factors for each kVp applied, as shown in Table S1 of the Supplementary material.

**Table 1:** Conversion factors for kVp-independent PCAT_MA_ evaluation according to scanner manufacturer and tube voltage. Conversion factors were calculated using Eq. (1) provided in the text and derived from the IR datasets

	Tube voltage (kVp)			
	80	100	120	140
256-slice CT	1.292 ± 0.086	1.049 ± 0.088	1 (reference)	0.923 ± 0.030
Dual-source CT	1.241 ± 0.017	1.111 ± 0.042	1 (reference)	0.970 ± 0.022
Average	1.267 ± 0.026	1.080 ± 0.031	1 (reference)	0.947 ± 0.024

*Abbreviations: CT* computed tomography, *IR* iterative reconstruction, *kVp* kilovoltage peak, *PCAT*_*MA*_ pericoronary adipose tissue mean attenuation

## Discussion

Our study is the first to report on the effect of tube voltage and IR on PCAT attenuation in a phantom study using ex vivo porcine hearts. The main findings of our study are as follows: (1) The contrast-enriched agar injections into coronary arteries of porcine hearts provide a usable CCTA phantom for PCAT_MA_ quantification. (2) Increasing tube voltages for CCTA acquisition were associated with higher PCAT_MA_ values. (3) The implementation of IR resulted in higher PCAT_MA_ values, as compared to conventional FBP reconstruction.

PCAT attenuation has recently received increased attention as a novel imaging biomarker due to its predictive value for cardiovascular outcomes [3, 7, 15]. Nevertheless, differences in acquisition protocols and reconstruction parameters may introduce bias in the analysis of PCAT attenuation by affecting CT numbers. For example, the tube voltage is often adapted in the clinical routine according to the patient’s characteristics and clinical questions. In addition, the use of low-kilovoltage CCTA has rapidly increased in recent years thanks to the technical advancements of CT scanners [16, 17]. Indeed, the mean photon energy of polychromatic x-ray beams generated by low tube voltage protocols is closer to the k-absorption edge (33 keV) of iodine. Thus, the photoelectric effect is enhanced, resulting in improved vessel-to-tissue contrast while reducing the radiation dose and the amount of contrast agent [18]. The significant increase of PCAT_MA_ observed with increasing kVp in our study is the direct result of the energy dependence of the underlying linear attenuation indices. These findings have been also confirmed by a recent phantom study, which investigated the spectral behavior of fat attenuation by using a photon-counting detector CT. In particular, the authors reported increasing PCAT attenuation values with increasing energy levels on virtual monoenergetic image reconstructions [19].

Owing to their ability to mitigate noise and provide diagnostic-quality CT images at lower radiation dose, IR algorithms have become the standard reconstruction technique, replacing the traditional FBP [20–22]. Therefore, we reported conversion factors limited to IR datasets. However, the attenuation differences between FBP and IR detected for both CT manufacturers highlight the importance of taking into consideration the reconstruction algorithm in the initial study design of studies investigating PCAT attenuation. While the results related to the tube voltage are more general, the results for IR are specific to the algorithms evaluated and cannot be generalized to other approaches or manufacturers. Similarly, since it has been previously demonstrated that reconstruction kernels influence CT numbers [8, 10], the results reported in this study cannot be transferred to scans reconstructed by using different filters.

Our study design allowed for the indirect evaluation of the effect of phantom positioning in the CT scanners on CT attenuation. Like the findings of a previous report [23], different positions of the hearts from the scanner isocenter resulted in variations of the CT numbers and, therefore, of the conversion factors at each tube voltage applied. Although these values were not calculated from the same heart imaged at different positions, this highlights the importance of accurate and consistent patients’ centering in CT at baseline and follow-up scans to avoid misdiagnosis.

Overall, the specific conversion factors for PCAT attenuation derived from our experiments are in agreement with existing literature values on pericardial adipose tissue [7] and PCAT attenuation [11]. However, in our study, PCAT values were relatively higher in comparison to those reported by Ma et al [11], thus leading to an increase in the relevant conversion factors, especially at low kVp values. While we report *k*_80_ = 1.267, the PCAT values reported by Ma et al would result in ${k}_{80}^{\prime }=1.137$. On the other hand, Oikonomou et al used ${k}_{100}^{\prime }=1.11485$ and ${k}_{140}^{\prime }=0.89095$ based on attenuation values of pericardial adipose tissue [9], which are in line with *k*_100_ = 1.111 and *k*_140_ = 0.970, respectively, derived from our observations. The postmortem nature of our study, in combination with the injection of contrast-enriched agar and the lack of the surrounding anatomical structures, could potentially account for the observed shift in PCAT values at lower tube voltages.

Our study has some limitations. (1) Since the porcine hearts were obtained from the slaughterhouse, no detailed health information was available. Nevertheless, the hearts were generally considered healthy. (2) The phantoms were motionless, lacking motion artifacts that may occur during real-life CCTA scans. (3) Due to the ex vivo nature of the study, attenuation and beam hardening from chest structures expected in patients could not have been evaluated. Despite this, given the paucity of in vivo data on the effect of different tube energies on adipose tissue, we believe that our approach simulates better in vivo conditions than previous experiments limited to the use of tubes filled with oil. Since the aim of our study was to show the magnitude of the effect of tube voltage on CT numbers, rather than proposing conversion factors for the immediate implementation in clinical routine, it was deemed acceptable not to simulate additional absorptive and scattering effects from thoracic structures. (4) Although a minimal delay in the scanning of the hearts after preparation was ensured, diffusive effects might have propagated the contrast agent into the perivascular tissue and caused an apparent dilation of the vessel, potentially leading to the exclusion of perivascular adipose tissue during segmentation. Since the co-registration of the images ensured direct comparability of PCAT_MA_ at different tube voltages, the effect on the results should be negligible. (5) Despite preliminary data from human studies showing that body mass index and cardiovascular risk factors might have an impact on PCAT_MA_ [24], our study design did not allow the assessment of these clinical factors.

In conclusion, our study showed that PCAT_MA_ values vary considerably by tube voltage and reconstruction type. Therefore, standardized acquisition and reconstruction protocols are advisable to assure accurate, reproducible, and comparable PCAT attenuation values in multicenter and longitudinal studies.

## Supplementary Information

ESM 1 (DOCX 15.2 kb)

## Abbreviations

ADMIRE: Advanced modeled iterative reconstruction
ASIR-V: Adaptive statistical iterative reconstruction
CCTA: Coronary computed tomography angiography
CT: Computed tomography
ECG: Electrocardiogram
FBP: Filtered back projection
HU: Hounsfield unit
IR: Iterative reconstruction
kVp: Kilovoltage peak
LAD: Left anterior descending artery
LV: Left ventricle
mAs: Milliampere-seconds
PCAT: Pericoronary adipose tissue
PCAT_MA_: Pericoronary adipose tissue mean attenuation
RCA: Right coronary artery
RV: Right ventricle
VOI: Volume of interest

## Declarations

### Guarantor

The scientific guarantor of this publication is Professor Catherine Gebhard.

### Statistics and biometry

No complex statistical methods were necessary for this paper.

### Methodology

• prospective

• experimental

• multicenter study

## References

[1] Libby P, Tabas I, Fredman G, Fisher EA (2014). Inflammation and its resolution as determinants of acute coronary syndromes. Circ Res.

[2] Ridker PM, Everett BM, Thuren T (2017). Antiinflammatory therapy with canakinumab for atherosclerotic disease. N Engl J Med.

[3] Antonopoulos AS, Sanna F, Sabharwal N et al (2017) Detecting human coronary inflammation by imaging perivascular fat. Sci Transl Med 9(398):eaal2658 10.1126/scitranslmed.aal2658 28701474

[4] Lin A, Kolossvary M, Yuvaraj J (2020). Myocardial infarction associates with a distinct pericoronary adipose tissue radiomic phenotype: a prospective case-control study. JACC Cardiovasc Imaging.

[5] Lin A, Nerlekar N, Yuvaraj J (2021). Pericoronary adipose tissue computed tomography attenuation distinguishes different stages of coronary artery disease: a cross-sectional study. Eur Heart J Cardiovasc Imaging.

[6] Oikonomou EK, Antonopoulos AS, Schottlander D (2021). Standardized measurement of coronary inflammation using cardiovascular computed tomography: integration in clinical care as a prognostic medical device. Cardiovasc Res.

[7] Oikonomou EK, Marwan M, Desai MY (2018). Non-invasive detection of coronary inflammation using computed tomography and prediction of residual cardiovascular risk (the CRISP CT study): a post-hoc analysis of prospective outcome data. Lancet.

[8] Cademartiri F, La Grutta L, Runza G (2007). Influence of convolution filtering on coronary plaque attenuation values: observations in an ex vivo model of multislice computed tomography coronary angiography. Eur Radiol.

[9] Okayama S, Soeda T, Takami Y (2012). The influence of effective energy on computed tomography number depends on tissue characteristics in monoenergetic cardiac imaging. Radiol Res Pract.

[10] Achenbach S, Boehmer K, Pflederer T (2010). Influence of slice thickness and reconstruction kernel on the computed tomographic attenuation of coronary atherosclerotic plaque. J Cardiovasc Comput Tomogr.

[11] Ma R, Ties D, van Assen M (2020). Towards reference values of pericoronary adipose tissue attenuation: impact of coronary artery and tube voltage in coronary computed tomography angiography. Eur Radiol.

[12] Hunter J (2007). Matplotlib: A 2D graphics environment. Comput Sci Eng.

[13] Topp EJ (2001) SciPy: Open source scientific tools for Python. Available via https://www.scipy.org/. Accessed 7 Sep 2021

[14] Eun Sul Lee RNF (2006). Analyzing complex survey data.

[15] Bengs S, Haider A, Warnock GI (2021). Quantification of perivascular inflammation does not provide incremental prognostic value over myocardial perfusion imaging and calcium scoring. Eur J Nucl Med Mol Imaging.

[16] Stocker TJ, Deseive S, Leipsic J (2018). Reduction in radiation exposure in cardiovascular computed tomography imaging: results from the PROspective multicenter registry on radiaTion dose Estimates of cardiac CT angIOgraphy iN daily practice in 2017 (PROTECTION VI). Eur Heart J.

[17] Stocker TJ, Leipsic J, Hadamitzky M (2020). Application of low tube potentials in CCTA: results from the PROTECTION VI Study. JACC Cardiovasc Imaging.

[18] Meyer M, Haubenreisser H, Schoepf UJ (2014). Closing in on the K edge: coronary CT angiography at 100, 80, and 70 kV-initial comparison of a second- versus a third-generation dual-source CT system. Radiology.

[19] Mergen V, Ried E, Allmendinger T (2022). Epicardial adipose tissue attenuation and fat attenuation index: phantom study and in vivo measurements with photon-counting detector CT. AJR Am J Roentgenol.

[20] Den Harder AM, Willemink MJ, De Ruiter QM (2016). Dose reduction with iterative reconstruction for coronary CT angiography: a systematic review and meta-analysis. Br J Radiol.

[21] Utsunomiya D, Weigold WG, Weissman G, Taylor AJ (2012). Effect of hybrid iterative reconstruction technique on quantitative and qualitative image analysis at 256-slice prospective gating cardiac CT. Eur Radiol.

[22] Mohammadinejad P, Mileto A, Yu L (2021). CT noise-reduction methods for lower-dose scanning: strengths and weaknesses of iterative reconstruction algorithms and new techniques. Radiographics.

[23] Szczykutowicz TP, DuPlissis A, Pickhardt PJ (2017). Variation in CT number and image noise uniformity according to patient positioning in MDCT. AJR Am J Roentgenol.

[24] Hell MM, Achenbach S, Schuhbaeck A, Klinghammer L, May MS, Marwan M (2016). CT-based analysis of pericoronary adipose tissue density: relation to cardiovascular risk factors and epicardial adipose tissue volume. J Cardiovasc Comput Tomogr.

