# Supplementary material for: Towards universal comparability of pericoronary adipose tissue attenuation: a coronary computed tomography angiography phantom study
Source: Eur Radiol. 2022 Dec 6;33(4):2324–30. doi: 10.1007/s00330-022-09274-5 (PMC10017558; doi:10.1007/s00330-022-09274-5)
Supplement: Supplementary file 1 — (DOCX 15.2 kb) [file 330_2022_9274_MOESM1_ESM.docx]

**Table S1. Tube voltage-specific conversion factors for the different positions of the hearts inside the phantom.**

|  | **80kVp** | **100kVp** | **120kVp** | **140kVp** |
| --- | --- | --- | --- | --- |
| **256-slice CT** | | | | |
| **Top row**  **(centered position)** | 1.295 | 1.047 | 1 | 0.941 |
| **Left bottom row** | 1.235 | 1.032 | 1 | 0.924 |
| **Right bottom row** | 1.407 | 1.088 | 1 | 0.880 |
| **Dual-source CT** | | | | |
| **Top row**  **(centered position)** | 1.232 | 1.060 | 1 | 0.992 |
| **Left bottom row** | 1.227 | 1.156 | 1 | 0.975 |
| **Right bottom row** | 1.267 | 1.127 | 1 | 0.939 |

*Abbreviations: CT, computed tomography; IR, iterative reconstruction; kVp, kilovoltage peak; PCAT_MA_, pericoronary adipose tissue mean attenuation*
